# How much less is enough? A reflexive perspective on the theory of simplified information processing by Voigt et al. (2025)

**DOI:** 10.3389/fsoc.2026.1796558

**Published:** 2026-09-18

**Authors:** Swen Koerner, Mario S. Staller

**Affiliations:** 1 Department of Training Pedagogy and Martial Research, German Sports University Cologne, Cologne, Germany; 2 Department of Police, University for Police and Public Administration North Rhine-Westphalia, Cologne, Germany

**Keywords:** decision-making, motor heuristics, movement analogies, reflexivity, replication crisis, second order observation, systems theory, theory crisis

## 1 Introduction

Voigt et al. (2025) propose a unified theory of simplified information processing during sportive actions by integrating two concepts: motor heuristics (decision strategies) and movement analogies (execution strategies). Their central argument is that both constructs fulfill functionally equivalent roles—namely, reducing cognitive load under performance pressure—and can therefore be subsumed under a shared framework of bounded rationality. The aim is a theoretically parsimonious yet empirically viable account that does not treat decision and execution as separate research domains, but as variants of a general principle of simplification. Importantly, this integration is not merely additive. Voigt et al. (2025, p. 5) explicitly claim a conceptual unification (“unifying framework”) and frame both concepts as “functionally equivalent” strategies. The added value thus lies not in “more theory” in the sense of accumulating heterogeneous explanations, but in “more unity”: theoretical complexity is reduced by relating different means—motor heuristics and movement analogies—to a shared function, namely simplified information processing. We explicitly acknowledge this achievement.

Our commentary therefore does not target the ambition to integrate, but its epistemic preconditions: From our perspective, “functional equivalence” is not a property simply “contained in behavior.” It depends on which function is observed, which timescales are selected, which contextual dimensions are treated as relevant—and what is excluded as irrelevant “noise.” The key issue is thus not only whether the integration works, but under which observational assumptions it can appear as successful. We propose a reflexive perspective that shifts the focus: away from the question of what decisions are and toward the question of how decisions become observable as such. Building on systems-theoretical observation theory (Luhmann, 2012; Baecker, 1999), we argue that a model's strength lies not only in its empirical reach but also in the observational logic it employs. This perspective does not reject integration; rather, it embeds it within a broader epistemological understanding that renders the constructive logic of theoretical distinctions visible.

## 2 Integration as theoretical added value

Voigt et al.'s (2025) proposal rests on a central insight: although motor heuristics (Gigerenzer and Gaissmaier, 2011) and movement analogies (Schücker et al., 2013) originate from different research strands, they play functionally similar roles in reducing complexity—primarily in action selection in the former case and in action execution in the latter. Integration thus bridges cognitive and motor levels and provides a shared rule vocabulary for describing simplification under time pressure. This unification is further sharpened by Voigt et al.'s (2025, p. 3) explicit coupling of their integration programme to an empirical guiding norm: theoretical integration should start from “concrete phenomena, defined as replicable empirical observations or empirical generalizations”. This also highlights the dual meaning of their guiding motto “less is more”: practically, it refers to simplification as an advantage for action (more efficient deciding/executing); theoretically, it refers to unification as a reduction of theoretical complexity.

The programme is framed within a classic information-processing structure: stimuli (input) are filtered by cognitive mechanisms (selection) and result in observable behavior (output). This linear logic resonates with canonical models of cognitive psychology (Newell and Simon, 1972; Simon, 1976) and allows for high operationalizability. This is a clear advantage: the theory enables measurable, replicable, and communicable claims about decision and execution behavior. Yet the very formal precision and empirical tractability also mark a boundary: what becomes visible is already shaped by the decision to model action as information processing.

## 3 From first order modeling to second order observation

Voigt et al.'s integration is grounded in a basic theoretical decision: action is structured as cognitive processing. This decision enables comparison and combination of both constructs within a shared rule system. The epistemic value lies in internal consistency—but also in stabilizing distinctions that define what counts as “decision,” “action,” or “simplification.”

From a systems-theoretical perspective, every theory denotes something within the frame of a distinction (Luhmann, 2012). With every designation come implicit assumptions and exclusion zones: what falls inside the chosen distinction becomes visible, while other aspects appear as background or “noise.” They remain unobservable. Second-order observation begins precisely here: it observes not only the object (decision behavior) but also the distinctions through which that object is constituted as such. In systems-theoretical terms, the observational logic of a theory can be read particularly clearly from the concepts it presupposes. Voigt et al. operate with indications such as “decision,” “action,” and “information.” Each of these indications is possible only within the frame of a distinction: “decision” presupposes a difference from non-decision (or from reaction/routine); “action” presupposes a difference from mere behavior or occurrence; “information” presupposes a difference from non-information (or noise). These distinctions entail implicit assumptions—for instance, the assumption of attributable agency: a subject (or attributable instance) must be presupposed to which selection, action units, and information processing can be ascribed.

Second-order observation does not only ask how decisions “function,” but through what operations they appear as decisions in the first place. It reconstructs the theory's logic of connectability—that is, which descriptions become connectable (e.g., input–filter–output, selection/execution, information/noise) and which possibilities remain invisible (e.g., decision as emergent coupling, action as continuous process, information as a system-relational product of selection). Reflexivity in this context means making visible the observational decisions that determine what can count as a factor at all (Koerner and Staller, 2022).

In this sense, the claimed unity of heuristics and analogies is itself the result of an observational decision. “Functional equivalence” is not a property of action independent of theory, but an attribution within a framework that codes simplification as a cognitive gain. Reflexively, the point is: the integration works because an observational logic has already been set that describes selection and execution as two addressable sub-problems of the same information-processing system—and treats this segmentation as scientifically productive.

When Voigt et al. tie integration to replicable effects, this implies that replicability validates the theory. From a reflexive perspective, however, replicability first and foremost confirms the stability of the categories and measurement routines that define what counts as an effect. The replication crisis (Open Science Collaboration, 2015) and diagnoses of an accompanying theory crisis (Eronen and Bringmann, 2021; Green, 2021; Oberauer and Lewandowsky, 2019) suggest that stability should not automatically be equated with adequacy to the object. Replication can equally indicate that an observational form (operationalizations, designs, measurement logics) reproduces itself reliably—regardless of whether the observed object is “in itself” stable.

## 4 Observing information and decision

The central concept in Voigt et al.'s model is information: something is observed, filtered, interpreted, and implemented. Yet from a systems-theoretical perspective, “information” is not an objective property of the world—it arises only within a system that distinguishes signal from noise (Luhmann, 2012). Information is thus not a substance “out there,” but a form generated in observation: a difference is marked as a difference and made connectable.

This has implications for decision making. What counts as “decision-relevant” is the result of an observational logic that codes stimuli as input, assumes cognitive processing as a filter, and describes behavior as output. Within this logic, decision appears as the operation of an agent who compares alternatives and selects. This semantic makes decision empirically manageable—but it is highly presuppositional. It presupposes, among other things: (a) a reconstructable set of alternatives, (b) an attribution of “selection” as a distinct process, and (c) the possibility of analytically separating selection and execution components.

Voigt et al. explain how decisions are simplified; they ask less under which assumptions something appears as a “decision.” From a reflexive perspective, this is a consequence of the observational form: once decision is posited as a psychological unit, one can ask for more efficient rules without thematizing the very assumptions that make “decision” visible as a unit in the first place. This is precisely the added value of second-order observation: it shows that the object (decision) is not merely described but is co-produced through theoretical distinctions.

That objects can be observed differently is illustrated by ecological dynamics (Davids et al., 2013). Here, action is often modeled as an emergent interplay of body, perception, and environment without presupposing a strict decomposition into selection and execution. Our point here is not that this approach is “more objective,” but that the same object is contoured differently depending on which distinctions are used. This pluralism makes visible that the segmentation into selection/execution is not natural but an observational product—and thus, in principle, variable and thus contingent.

## 5 Reflexivity as a resource

Voigt et al. (2025, p. 4) link their integration programme to replicable empirical phenomena. From a reflexive perspective, however, replicability is not an immediate proof of truth but an indicator that categories, instruments, and routines function consistently (Staller and Koerner, 2026). In a crisis widely discussed as both a replication and theory crisis, this insight can become productive: replicability is not treated as an endpoint but as an occasion to examine the conditions under which stability can reasonably be expected.

This is also the central point with respect to the implicit progress semantics that may accompany theoretical integration. Voigt et al. formulate their project as a descriptive programme; they intend to understand strategies rather than prescribe them. Nevertheless, an implicit normativity can arise from the selection of descriptive categories itself: if simplification is primarily read as cognitive efficiency, “less is more” appears not only as a practical rule for action but also as an epistemic ideal of reductive explanation. Normativity then emerges less as intention than as an effect of what becomes plausible and “well explainable” within the chosen observational logic.

For empirical research practice, reflexivity is no luxury. It can function as a methodological corrective that makes explicit the observational decisions embedded in design choices, variable selection, and operationalization. If a “decision” does not appear experimentally as expected, this need not primarily indicate “poor performance”; it may signal that the chosen distinction (decision vs. non-decision; relevant vs. irrelevant information) does not hold in the situation. Reflexivity allows such discrepancies to be treated not only as confounds but also as indicators of categorical misfit—and thus as epistemic opportunities (Uher et al., 2025).

From this perspective, replication itself can become a second-order instrument: not only for confirmation but for mapping boundary conditions. From our perspective, productive research is not limited to repetition under identical conditions; it also includes controlled variation of the observational form (e.g., alternative operationalizations of “information,” different segmentations of action trajectories) to examine which stabilities are categorically produced and where the phenomenon resists measurability.

The following example may illustrate this shift for empirical research on decision-making in sport. A conventional replication study might test whether athletes under time pressure rely on fewer cues and whether this improves response speed or accuracy. In such a design, “information” is typically operationalized as externally defined cue density, “decision” as the selection of one option, and “performance” as the adequacy or speed of the selected response. A reflexive extension, which could be called “second order replicability,” would vary these observational categories. The same situation could be reconstructed once as a discrete selection problem, once as an unfolding perception-action coupling, and once as a situation in which cue relevance is determined by the athlete's own subjective significance. The empirical question would then change: not only whether simplified information processing can be replicated, but under which categorical description the effect changes.

Second-order replicability can therefore be specified as a methodological extension of conventional replication. Conventional replication asks whether an effect can be reproduced under comparable operational conditions. Second-order replicability asks how the reproducibility of an effect changes when the observational categories that constitute the effect are systematically varied. Methodologically, this involves three steps: first, reproducing the finding within the original operational logic; second, varying key categories such as “information,” “decision,” or “action”; and third, comparing which empirical stabilities persist, disappear, or shift across these variations. Likewise, the status of the selection/execution distinction could be varied: once as strictly separated phases (compatible with input–filter–output), once as continuous reciprocal adaptation (compatible with ecological dynamics). Empirically, the question would then not be “which model wins,” but which stabilities occur under which observational decisions.

Through this lens, we do not intend to weaken the benefits of replication. Yet we see value in making its epistemological conditions visible and thus shift what replicability can achieve: it indicates stability within an observational form whose scope, limits, and costs can systematically be examined.

## 6 Discussion: How much “less” is enough?

Voigt et al. provide a strong, empirically connectable model for explaining simplified decision and execution processes. Their theoretical contribution lies in the conceptual unification of two previously separate logics of simplification (“unifying framework,” “functionally equivalent”; Voigt et al., 2025, p. 3) and in explicitly linking this unification to replicable empirical phenomena (Voigt et al., 2025, p. 4). Precisely for this reason, the paper is an ideal starting point for observing the observational conditions of such unification: which distinctions (information/noise, selection/execution, simplification/complexity) must be made for the theory to unfold its strengths, and which alternatives thereby become systematically less visible?

Our commentary therefore argues for a shift: away from mere accumulation of theories and toward observation of theorizing itself. The slogan “less is more” thus gains a dual meaning: practically, as the advantage of simplified rules in action; theoretically, as an ideal of reductive explanation. Second-order observation makes visible that “less” is not only a training heuristic but also a scientific programme—associated with gains (operationalizability, comparability, replication) and costs (selectivity, blind spots).

The title question—how much “less” is enough?—thus points to a tension: reduction produces effects of objectivity because it enables measurement and replicability, yet the same reduction generates exclusion zones. Form a systems-theoretical perspective, this objectivity is not a position outside observation, but an operational achievement dependent on specific operations of observation, distinction and stabilization. The scientific value of reflexive perspectives does not lie in abandoning objectivity, but in making its conditions explicit and thereby specifying the reach of models more precisely. In light of the replication and theory crisis, this gain in transparency may constitute the decisive form of progress: not (only) more models, but greater awareness of modeling itself.
